# Rush Medical College

**Published:** 1860-12

**Authors:** 


### RUSH MEDICAL COLLEGE.

The friends of this institution will be gratified to hear of its continued high prosperity. During the present session it numbers a much larger class than at any previous one since its organization. This is true both with reference to the list of under-graduates and of practitioners in attendance.

A contemporary journal publishes, as an item of news, that *seventy-five* students were present at the opening of the course! If it had put the number at double this, it would have approximated the figure more closely. The next ensuing catalogue will exhibit the fact that the present large class of Rush Medical College is composed of *bona fide* medical students, and not, as in at least one instance we wot of, of *invited* hearers from the classes of the various *Theological Seminaries*, and employees of every variety in the crafts and trades of the town and suburbs, almost "compelled to come in," to inflate an *unpublished*, because barren, catalogue.

It is barely possible that the spirit of petty spleen, which prompted the item alluded to above, may, if persistently manifested, secure a ventilation of the facts, which it were well for that writer to avoid—at least until after the next June meeting of the National Medical Association.

The Faculty of the College have devoted their best energies and most unsparing industry to the work of developing the institution to a degree suitably commensurate with the field it occupies, and are already cheered and encouraged by the marked evidences of general professional confidence and support which they are receiving from the great North-West. The elements of opposition, which some time since awakened the apprehension of the distant *alumni* of the College, have already, in effect, disappeared, and a career of high usefulness and honor seems cloudless in the future.

